# Improvement of Flow Velocity Measurement Algorithms Based on Correlation Function and Twin Plane Electrical Capacitance Tomography

**DOI:** 10.3390/s20010306

**Published:** 2020-01-06

**Authors:** Volodymyr Mosorov, Marcin Zych, Robert Hanus, Dominik Sankowski, Ayoub Saoud

**Affiliations:** 1 Institute of Applied Computer Science, Lodz University of Technology, 90-537 Lodz, Poland; dsan@kis.p.lodz.pl; 2 Faculty of Geology, Geophysics and Environmental Protection, AGH University of Science and Technology, 30-059 Kraków, Poland; zych@geol.agh.edu.pl; 3 Faculty of Electrical and Computer Engineering, Rzeszów University of Technology, 35-959 Rzeszów, Poland; rohan@prz.edu.pl; 4 Infosys Poland Ltd., 91-402 Lodz, Poland; saoud_ay@yahoo.com

**Keywords:** electrical capacitance tomography, signal pattern, cross-correlation, plug regime, time window

## Abstract

This article discusses the correlation method for time delay estimation, its disadvantages, and drawbacks. It is shown that the correlation method for material velocity measurement based on images of instantaneous changes of the concentration material inside measured by twin planes electrical tomography has serious limitations, especially in the case of plug regime. The basic problem is the non-stationarity of measured data, therefore the requirement of *correlability* of input data should be fulfilled. The requirement *correlatability* of input data imposes limitations on the possibility of continuous velocity measurement. This means that the material velocity can only be calculated when data are correlatable. An original algorithm of automatic extraction of the suitable time intervals to calculate material velocity is proposed. The algorithm allows measuring the flow velocity in a proper and accurate way. The examples of the correct velocity calculation, using the proposed concept for the gas-solid flow regime, are presented.

## 1. Introduction

Currently, the transportation of granular material is widely used in various applications, like mining (e.g., mining of polymetallic nodules), mineral, chemical, and pharmaceutical industries [1,2,3,4,5]. Elaboration of a low-cost pneumatic transport system requires taking into consideration the influence of such parameters like velocity, material distribution, flow regime, etc., on its transport capacity. Maintaining low air pressure during granular material propagation is one of the basic criterions of the efficiency of such a transportation system. Therefore, real-time measurement of solid velocity in pneumatic transport can be useful for the improvement of the transportation efficiency of granular material. Nowadays several different techniques for flow velocity measurement are used in practice although they have many shortcomings. Thus, invasive techniques require sensors inserted into the pipe and as a result, they disturb the material propagation and are exposed to abrasion over time. Laser and other optical methods have a limited scope of use, whereas safety standards must be considered in the case of the absorption techniques, which is based on radiation measurements. 

To design the flow meters, several non-invasive techniques are available. For instance, displacement-current phase tomography (DCPT) is a process tomographic technique originally proposed for two-phase flows where one phase is electrically conductive, such as water [6]. When flow phases are not electrically conductive, electrical capacitance tomography (ECT) technique will be applied. The classical tomographic image velocimeter (TIV) includes twin sensor planes mounted around an investigated pipe/vessel/column. Each sensor consists of electrodes, (typically a number of the electrodes is 8, 12, 24) and it allows measuring the electrical capacitance between any pairs. Next, an image reconstruction algorithm is used for visualization of the material distribution inside the investigated volume based on these capacitance measurements. To calculate material velocity at the sensors planes, a series of reconstructed images are required. 

Although many papers concerning the designing of flow meters based on the ECT technique have been published in the last decades, the correctness and reliability of a particle velocity measurement of solid-gas flow are a key challenge [7,8]. Contrary to the alternative techniques based on the computation of the sensitivity matrix spatial gradient [6,9], the classical cross-correlation method [10,11,12,13,14] uses transit time to estimate material velocity. If we use the correlation technique, in general, the transit time corresponds to a time of a peak of the cross-correlation function of signals representing material distribution changes within a given pixel of the first and second plane of the tomographic unit. It must be emphasized that the correlation function is calculated at the chosen time window. This time interval specifies the number of images that are considered to calculate the correlation function. Generally, the length of such a time window is arbitrarily chosen as the fixed value and there are no explanations regarding its determination [15,16,17,18]. However, the length of the time window has a strong influence on velocity calculations. Thus, the time window must comprise suitable *signal patterns* extracted from plugs or slugs, which could be used for the material velocity calculation. Warsito and Fan [19] considered tracing flow structures, but they did not explain how to choose the suitable time windows. Fuchs et al. [20] described the slug-length determination method for a pneumatic conveying system, but again, they did not explain how we can determine the window length for the velocity calculation. Also, it is important to mention that the transit time is calculated for a time window; therefore, this calculated velocity corresponds to average material velocity between two sensor planes. 

The aim of the article is to fill up the existing gap by developing a new concept for an appropriative algorithm for particle velocity measurements using twin-plane electrical tomography. The proposed algorithm should automatically detect when it is possible to measure the particle velocity based on the obtained series of tomographic images. The authors suggest that the proposed algorithm can easily be applied to existing tomographic image velocimeters and for other modalities such as impedance or optical tomography.

## 2. Theoretical Considerations 

**Figure 1:**
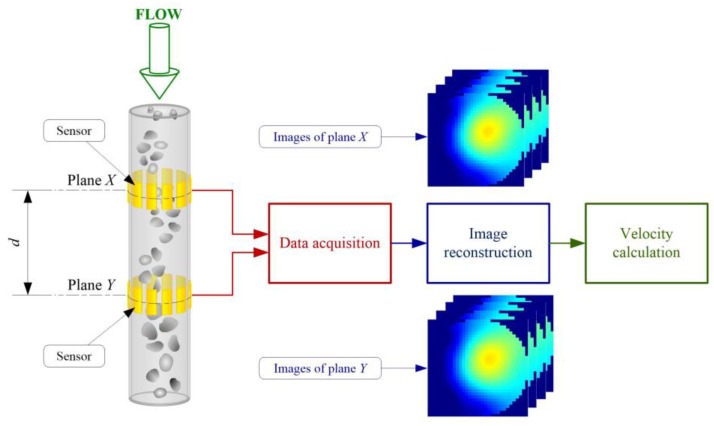
Material velocity measurement based on a twin plane electrical capacitance tomography (ECT) tomograph, *d* is the distance between two planes.

A twin plane ECT system to determine a material velocity distribution consists of two plane sensors (plane *X*, plane *Y*) which are mounted on a pipe (see Figure 1). The distance *d* between the two planes is known. The ECT tomograph provides images with the speed of imaging *T,* which represent the distribution of the material in cross-sections of the planes. In the case of solid-gas flow, such a distribution shows material concentrations at chosen time moments. 

As aforementioned, material velocity measurements achieved by the cross-correlation method are based on the determination of time delay. Let $x_{i,j}\left(nT\right)$ and $\;y_{i,j}\left(nT\right),\;i=0,\ldots ,I-1$, *j*= 0,…,*J*−1 define the material distribution changes for (i,j) pixel of the nth *I × J* pixels of tomograms (see Figure 1) with the frame rate resolution  T obtained from planes X and Y of the twin plane tomograph, respectively.

When it is assumed that there is a strong relationship between the flow structure coming from the *X* plane and the ones coming from the *Y* plane and the cross-correlation function $R_{i,j}\left(kT\right)\;$ of two time series $\left\{x_{i,j}\left(nT\right)\right\}$ and $\;\left\{y_{i,j}\left(nT\right)\right\},\;$*n* = *N*,…*N* + *M −* 1 containing M observations from the plane X and Y can be calculated as follows:

(1) $$R_{i,j}\left(kT\right)=\frac{1}{M}\overset{N+M-1}{\underset{n=N}{\sum }}x_{i,j}\left(nT\right)y_{i,j}\left(\left(n-k\right)T\right),\;\;k=\cdots ,-1,0,1\ldots$$

The cross-correlation maximum *R_max_* corresponds to the lagged time $\tau_{0}=pT$ between the two time series. The average velocity $\bar{V}$ of the flow in (*i*, *j*) pixel of the cross-section calculated in time window [*N*, *N*+*M*] is then given by the following:

(2) Vi,j¯= dτ0

where d is the distance between the two tomographic sensors.

**Figure 2:**
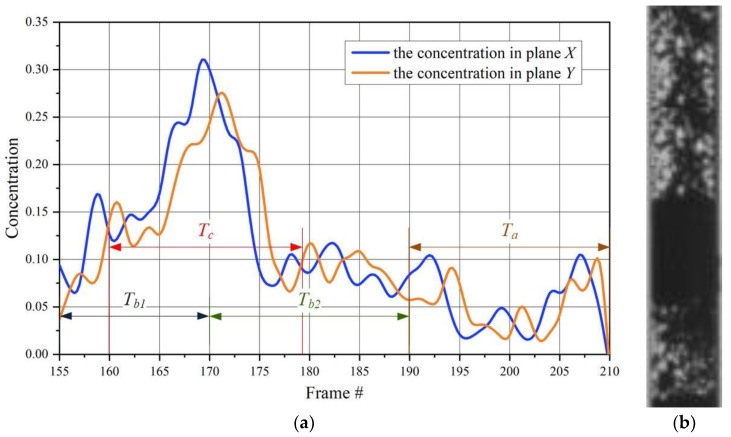
Normalized concentration changes within chosen pixel (12,12) of the tomographic images (**a**) and image captured by high-speed camera (#150-#205) (**b**); *T_a/b/c_*—the fixed time window.

Figure 2 shows the example of typical normalized concentration changes (i.e., 0 means an empty pipe and 1 means a pipe filled up material) in the chosen pixel of the tomographic images captured by a twin plane ECT tomograph for a pneumatic conveying flow in a vertical pipe section during the plug propagation. As have been mentioned before, the estimation of the time lags between two-time series is based on the determination of the global peak of a cross-correlation function. It is obvious that such a peak can incorrectly be determined for the strong noised input signals. The problem can be solved by improvement of measurement unit parameters. Additionally, the correlatability will be dependent not only on the type and regime of flow, but also on the spatial separation *d* between the measurement planes (large separations will naturally decrease the correlatability). In this article, this problem has been also omitted. Another basic problem is whether such a global maximum for the calculated correlation function exists. In practice, two cases can appear: The correlation function cannot have an evident peak i.e., it is whether an increasing or decreasing function and the second one is that the correlation function may have many local maxima only.

Considering the time series representing concentration changes in the chosen image pixels captured by sensor planes *X* and *Y* (see Figure 2) and assuming that time window is fixed and moved, the following time intervals *T_a/b/c_*, could be used for correlation calculation:

1. There are no moving structures inside a pipe (material is absent or its concentration is too small), the local concentrations represent noise signals during the chosen time interval, e.g., *T_a_*. This means that there is no sense to calculate the cross-correlation, and velocity cannot be determined.
2. The chosen time window comprises the beginning/ending of an upcoming plug, for instance, time intervals T_b1_ or T_b2_. In this case, there is also no possibility to determine the lag time because the calculated correlation function will have no peak.
3. The pixel’s changes from both planes X and Y have impulse shapes for instance interval Tc, and therefore they are suitable for cross-correlation function calculation. The peak of the correlation function will be evident.

**Figure 3:**
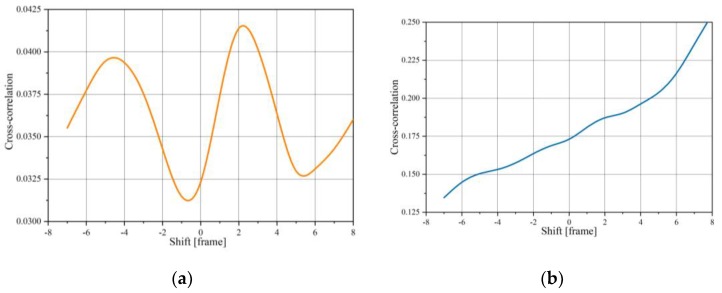

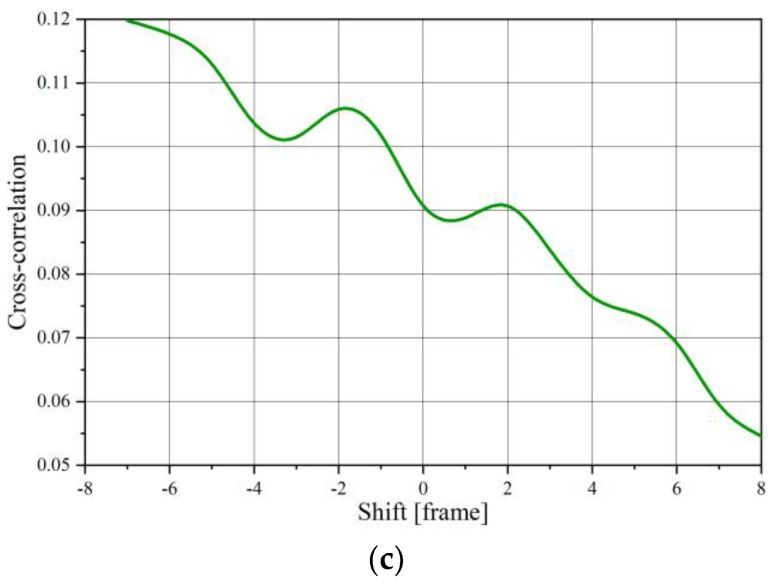
Examples of the correlation function of the normalized changes (see Figure 2) for different time windows *T* in frame: *T_a_* = [190–210] (**a**), *T_b_*_1_ = [150–170] (**b**), and *T_b_*_2_ = [170–190] (**c**).

Figure 3 shows the cross-correlation function calculations of two signals for the aforementioned time windows *T_a_*/*T_b_*. As can be seen, the calculated cross-correlation is not appropriate to lag determination. 

Hence, velocity calculation should consider choosing the appropriate signal patterns of time series $x_{i,j}\left(nT\right)$ and $y_{i,j}\left(nT\right)$ i.e., determination of a time window.

## 3. Adaptive Algorithm of Time Window Determination

To determine the corrected time window, appropriate signal patterns for which the cross-correlation function will have an evident peak should be required. In the case of flow propagation in the form of so-called plugs, for example, pneumatic conveying, signals representing changes in concentration, have shapes similar to a series of impulses occurring irregularly.

Each such “pulse” corresponds to the propagation time of one plug within the plane of the tomographic sensor. Since the plug propagation time is not constant, the choice of a time window with a fixed length *M* is not possible. In addition, plugs appear irregularly, therefore there is a problem: How to automatically determine the time of appearance of the plug and its finishing. Hence, the algorithm for determining the velocity based on the Equation (1) should automatically detect the moments of occurrence of the beginning of *N_b_* and the end *N_e_* of the plug.

Applying usual thresholding to determine the time moments is not possible due to the fact that there can be high probability of material occurrences in very short intervals of time. Therefore, the corresponding signals of changes in the pixel values in the reconstructed images will not be suitable for determining the material velocity by the correlation method due to their short duration. Such a case can be described as impulsive noise in terms of signal processing notions.

It is also obvious that the cross-correlation function cannot be directly calculated for *N_b_* and *N_e_* values in Equation (1). It is required to additionally add time intervals before and after to form a signal pattern to provide the same shape of a pulse. For the automatic determination of the appropriative time moments, which are useful to calculate the cross-correlation function, the following Algorithm 1 was proposed.
**Algorithm 1:** Time interval determinationChoose the threshold value s0 experimentally.Choose arbitrary an interval of confidence M0T, where M0 is the arbitrary chosen number of frames.Waiting when the signal $x_{i,j}\left(nT\right)$/$y_{i,j}\left(nT\right)$ is below the threshold value s0.If the signal value $x_{i,j}\left(nT\right)/y_{i,j}\left(nT\right)$ exceeds the threshold value s0 during the confidence interval [NbT, NbT+M0T] then moment NbT-M0T determines beginning of signal pattern.If the signal value $x_{i,j}\left(nT\right)$/$y_{i,j}\left(nT\right)$ is below the threshold value s0 during the interval of confidence [NeT, NeT+M0T] then moment NeT+M0T is chosen as the end of the signal pattern.The cross correlation function is calculated as the following:$$R_{i,j}\left(kT\right)=\frac{1}{N_{e}-N_{b}+2M_{0}}\overset{N_{e}+M_{0}}{\underset{n=N_{b}-M_{0}}{\sum }}x_{i,j}\left(nT\right)y_{i,j}\left(\left(n-k\right)T\right),\;k=\ldots ,-1,0,1\ldots \;$$Finally, velocity of the flow in (i,j) pixel is calculated according to Equation (2), however adding the interval [NeT, NeT+M0T]: Vi,j|[NeT,NeT+M0T]¯= dτ0Return to step 3 where n=Ne i.e., starting from the moment of the interval when the signal $x_{i,j}\left(N_{e}T\right)$/$y_{i,j}\left(N_{e}T\right)$. is below the threshold value s0.

**Figure 4:**
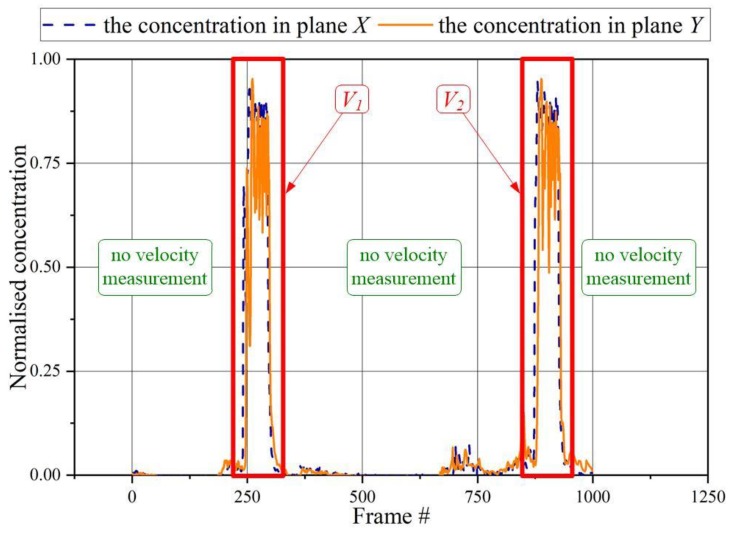
Examples of particle velocities calculation ability based on the proposed approach (arbitrary chosen threshold *s_0_=*0.1). The normalized concentration in the chosen pixel (15,15).

It should be emphasized that this approach assumes that the velocity is determined for specified signal patterns and corresponds to the material velocity in the specified time interval. To calculate the next speed value, it is required to find the next appropriate signal pattern. Figure 4 shows an example of concentration changes in a specific pixel for gas-solid flow in pneumatic transport (vertical section). As we can see, it is possible to measure the speed for two signal patterns only. Apart from these intervals, the particle velocity cannot be determined due to the low level of signals oscillating within the noise level. Alternatively, a long time interval can be applied, for instance from #201 to #955, however particle velocity measurement rate will fall. 

## 4. Experiments and Results

**Figure 5:**
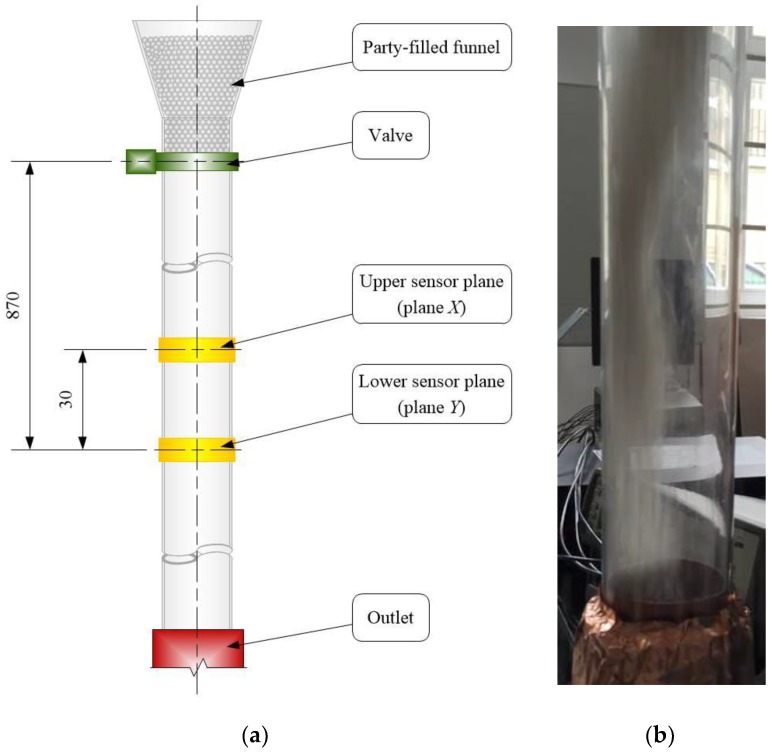
Gravity drop rig schematic (**a**), image of gravity flow (above upper sensor plane) (**b**).

Firstly, the proposed concept has been verified for a gravity flow process. The gravity drop rig schematic is shown in Figure 5. The measured pipe section with an inner and outer *D* 10 cm 12 cm, respectively, was filled with plastic pellets with a 3 cm diameter, and its density was 905 kg/m^3^. The particles were retained by a valve above the sensor planes. The 16 channels electrical capacitance tomograph made by ECT Instruments Ltd with data acquisition of 100 frames/s of each sensor was applied to the particle velocity measurement [21]. The ECT system had been calibrated for two cases i.e., empty and next fulfilling by particles to give a concentration range from 0 to 1.

**Figure 6:**
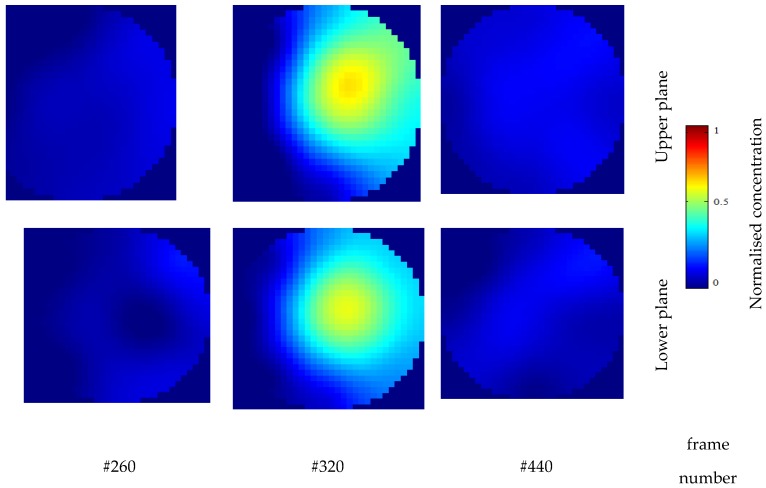
32 × 32 pixel ECT tomographic images of upper and lower plane for chosen frame number.

Figure 6 presents the examples of ECT normalized tomograms of two planes for chosen frames. The iterative back-projection algorithm with a fixed number of iterations 10 and the value of relaxation factor 1.2 was utilized for the image reconstruction [22,23].

**Figure 7:**
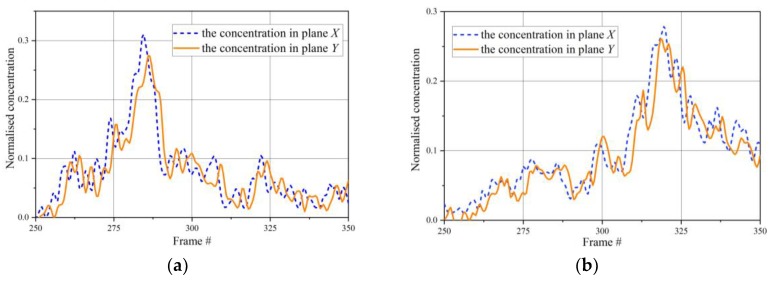

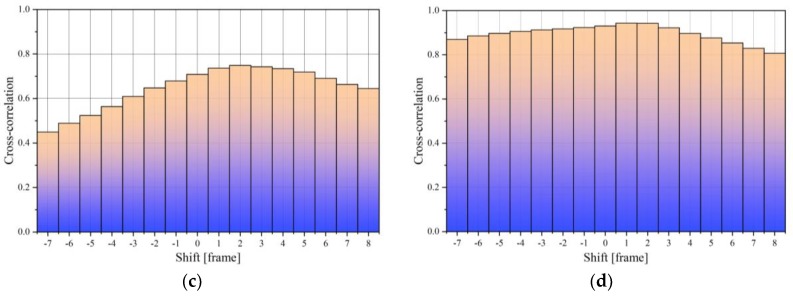
(**a**) Normalized concentration of the chosen pixel (15,15), (**b**) appropriative cross correlation functions at specified time intervals [#270, #305] and [#303, #345] by the proposed algorithm; (**c**,**d**) appropriate cross- correlation functions.

Figure 7a,b presents the examples of normalized concentration instantaneities in (15,15) pixel during material propagation through the pipe. Figure 7c,d shows the cross-correlation functions of the normalized concentration instantaneities (Figure 7a,b) in chosen pixel position at specified time intervals [#270, #305] and [#303, #345] by the proposed algorithm. The arbitrarily chosen threshold *s_0_* is 0.1 and the interval of confidence *M*_0_ is 5 frames. The maximum of the correlation function was at shift *p* = 2 for concentration changes representing in Figure 7a,b, respectively, and calculated particle velocity was 0.01 × 2/0.03 ≈ 0.667m/s.

To verify the proposed algorithm, a velocity *v_T_* of the fallen particles at the lower plane of the sensor was evaluated as follows [10]:

(3) $$v_{T}(iT)=v_{T}((i-1)T)+\left(g-\frac{C_{d}\cdot \rho_{a}\cdot A}{2\cdot m}v_{T}^{2}((i-1)T)\right)\cdot T,\begin{array}{cc} & v_{T}(0)=0,\begin{array}{cc} & i=1,\ldots \end{array} \end{array}$$

where *m* is the mass of the particle (1.3 × 10^−5^ kg), *g* is the gravity acceleration (9.81 m/s^2^), *C_d_* is the drag coefficient (0.5), *A* is the particle frontal area (7 × 10^−6^ m^2^), and *ρ_a_* is the air density (1.3 kg/m^3^). 

**Figure 8:**
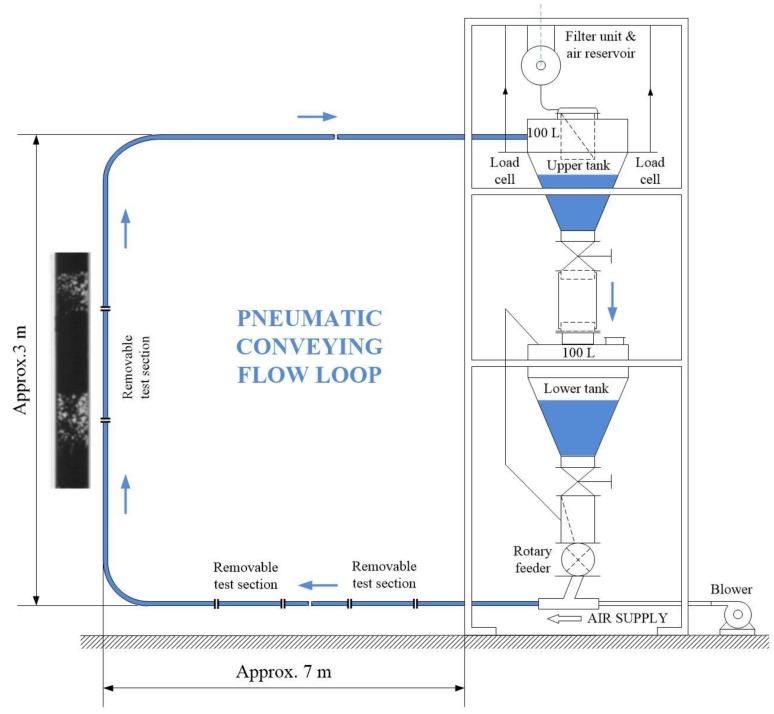
Gravity drop rig schematic (**right**) and image captured by a CCD camera installed closed to transparent removable test section (**left**).

According to Equation (3), particle velocity at the lower plane level reaches the value 0.721 m/s. Next, the proposed concept has been tested using the series of the reconstructed images captured by a twin-plane ECT tomography system PTL 300 (Process Tomography Ltd. 2000, Manchester, UK) with a frame rate of 100 frames/s. The full description of the pneumatic conveying flow rig (see Figure 8) is given in the study by Jaworski and Dyakowski [24]. Two sensors were mounted on the vertical pipe section. Figure 4 shows the concentration changes for the central pixel of the cross-section as the function of a frame number for an upward flow of plastic beads at an average rate of 900 kg/h blown by air at a superficial velocity of 2.2 m/s in a 50 mm ID pipeline. 

As seen from Figure 4, a plug appears between the 200th and 302nd frame and between the 850th and the 950th frame for image pixel (15,15). The time intervals were chosen according to the proposed algorithm at threshold value *s*_0_ = 0.05. 

The difference between velocity values obtained by the classical method and the proposed one causes a difference between the mass flow rate calculated. The mass flow rate of the suitable signal patterns $F_{pattern}$ was determined on the basis of velocity $v_{i,j,pattern}$ and average concentration $\bar{c_{i,j}}$ in (*i*, *j*) pixel of the tomographic image:

(4) $$F_{pattern}=\overset{I-1}{\underset{i=0}{\sum }}\overset{J-1}{\underset{j=0}{\sum }}\Delta S\cdot \hat{H\cdot }\rho \cdot v_{i,j,pattern}\cdot \bar{c_{i,j}}\;\;\;\;\;\;\;\;\;\;\;\left[kg/h\right]\;$$

where ρ is the bulk density of the material, $\hat{H}$ is the multiplier derived from the number of seconds in one hour, and ΔS is the area of a single pixel. 

In one of the experiments, the mass flow rate *F* equal to 715 kg/h was obtained for the classic correlation method with arbitrarily chosen time window 150 frames (time sampling *T* = 0.01 s), and around 746 kg/h for the proposed algorithm. The real solids flow rate obtained by the weighing method was 789 kg/h. In a series of 10 experiments, the standard deviation of the mass flow rate was 28% and 8.5% without/with considering the time intervals, respectively. The larger deviation value for the classical approach without considering the time intervals confirms the need to “synchronize” the time window with the appearance of a plug.

The open problem is the choice of the threshold value *s*_0_. The lower values of *s*_0_ allow one to determine more appropriative signal patterns, however it appears a risk wrong velocity calculation for weak detected signals. Therefore, the choice of level should be done experimentally for the concrete facilities.

## 5. Conclusions

Multiphase flow investigation and metering require a combination of multiple technologies, sensors, and algorithms in order to provide reliable and effective solutions. Known methods for transit time measurements do not consider the choice of time intervals in their calculations. This study gives a clear idea about how to calculate the velocity in the right way and also the ability to calculate the velocity for flow pattern tracking using the cross-correlation technique. Contrary to classical methods, the proposed concept considers an additional criterion, that is, the presence of flow patterns within the sensor volume. The developed signal pattern dependence method enables one to calculate transit time more appropriative, and in this way, to increase the scale of the measurements. The proposed approach presents an algorithm of flow pattern analysis with, in addition, the cross-correlation technique, which refines the material velocity calculation. Results were obtained for gravity flow experiments and pneumatic conveying, although the proposed method can also be used for other flows with different tomographic modalities.
